# Left Ventricular Assist Device Effects on Metabolic Substrates in the Failing Heart

**DOI:** 10.1371/journal.pone.0060292

**Published:** 2013-04-01

**Authors:** Lindsay B. Weitzel, Amrut V. Ambardekar, Andreas Brieke, Joseph C. Cleveland, Natalie J. Serkova, Paul E. Wischmeyer, Brian D. Lowes

**Affiliations:** 1 Department of Anesthesiology, University of Colorado Anschutz Medical Campus, Aurora, Colorado, United States of America; 2 Department of Cardiology, University of Colorado Anschutz Medical Campus, Aurora, Colorado, United States of America; 3 Department of Surgery, University of Colorado Anschutz Medical Campus, Aurora, Colorado, United States of America; 4 Department of Cardiology, University of Nebraska Medical Center, Omaha, Nebraska, United States of America; University of Chicago, United States of America

## Abstract

**Background:**

Heart failure patients have inadequate nutritional intake and alterations in metabolism contributing to an overall energy depleted state. Left ventricular assist device (LVAD) support is a common and successful intervention in patients with end-stage heart failure. LVAD support leads to alterations in cardiac output, functional status, neurohormonal activity and transcriptional profiles but the effects of LVADs on myocardial metabolism are unknown. This study set out to measure cardiac metabolites in non-failing hearts, failing hearts, and hearts post-LVAD support.

**Methods:**

The study population consisted of 8 non-ischemic failing (at LVAD implant) and 8 post-LVAD hearts, plus 8 non-failing hearts obtained from the tissue bank at the University of Colorado. NMR spectroscopy was utilized to evaluate differences in myocardial energy substrates. Paired and non-paired t-tests were used to determine differences between the appropriate groups.

**Results:**

Glucose and lactate values both decreased from non-failing to failing hearts and increased again significantly in the (paired) post-LVAD hearts. Glutamine, alanine, and aromatic amino acids decreased from non-failing to failing hearts and did not change significantly post-LVAD. Total creatine and succinate decreased from non-failing to failing hearts and did not change significantly post-LVAD.

**Discussion:**

Measured metabolites related to glucose metabolism are diminished in failing hearts, but recovered their values post-LVAD. This differed from the amino acid levels, which decreased in heart failure but did not recover following LVAD. Creatine and the citric acid cycle intermediate succinate followed a similar pattern as the amino acid levels.

## Introduction

Patients with chronic heart failure often have inadequate intake of calories and protein and diminished energy availability [1]. Likewise, it has been hypothesized that the failing heart is in a chronic energy starved state [2], [3] [4] and that abnormalities in metabolism contribute to myocardial dysfunction. [5] There are also considerable data to support an obesity paradox in heart failure with BMI's between 30–35 associated with lower mortality than BMI's closer to 25 [6]. Together, these ideas would seem to argue that patients with heart failure are energy depleted and it is plausible they could benefit from targeted nutrition therapy. Historically, nutritional interventions in this population focus on limiting sodium intake and maximizing intake of minerals such as calcium and magnesium [7], while little is known about metabolic substrates and metabolism in the failing heart.

Left ventricular assist device (LVAD) placement is becoming increasingly common as a bridge to transplant and as destination therapy. These patients often suffer from significant malnutrition both pre- and post- device placement. These devices have been shown to improve quality of life and survival in patients with end-stage heart disease when compared to medical therapy. [8] Many cardiac parameters improve following LVAD implantation; however, little is known about how this device affects the nutritional and metabolic parameters of the unloaded left ventricle (LV).

For some patients, unloading of the LV by LVAD placement leads to improved function of the native LV, known as reverse remodeling. Noted effects of reverse remodeling on the LV include reductions in mass, end-diastolic diameter, and ejection fraction compared to pre-LVAD implantation [9]. The hypoperfusion characteristic of HF leads to systemic neurohormonal and cytokine alterations, many of which are also normalized by LVAD use [10]. Despite normalization of numerous cardiac and systemic parameters post-LVAD implantation, incidence of successful LVAD removal without transplant is low [10]. A number of parameters do not recover post- LVAD, including those involving the extracellular matrix (increased collagen content, cross-linking, and myocardial stiffness) [11]–[13] and those involving genes related to metabolism [14].

The aim of this study was to measure key metabolites related to nutrition and energy in the myocardium of patients suffering from heart failure, and compare these measures to the myocardial metabolism in healthy patients and in patients following LVAD therapy. Our hypothesis is that the failing heart will have lower levels of most key metabolites than the non-failing hearts; and some of these metabolites will return to healthy levels after LVAD placement. This study focuses on levels of metabolites that might be altered via nutritional intervention.

## Methods

### Patients and Cardiac Tissue

Ethics Statement: The protocol for the collection, storage, and analysis of tissue for this study was approved by the Colorado Multiple Institutional Review Board and is outlined in a previous publication [15]. Written consent was given by patients for their information to be stored and used for research purposes. Eight patients with end-stage non-ischemic cardiomyopathy prior to LVAD placement were recruited and LV tissue samples were collected and flash frozen at the time of LVAD implantation (pre-LVAD) and at the time of cardiac transplant (post-LVAD) from each individual. Eight donor hearts harvested for transplant, but unused for reasons unrelated to the heart were utilized as the non-failing control samples. The pre-LVAD tissue sample was a core sample from the LV apex, at the in-flow cannulation site of the LVAD. The post-LVAD tissue sample was similar in size and was taken from the explanted heart. All tissue samples were directly flash frozen in liquid nitrogen in the operating room and transported to a −80°C freezer. No cardioplegia solutions were utilized. Pre-LVAD samples experienced <10 seconds of ischemia time from removal of tissue from the patient to when it was placed in liquid nitrogen. For post-LVAD samples this time was <10 minutes.

Retrospective review of medical records for demographic and clinical data was conducted by a trained physician. Time points for echocardiographic and hemodynamic data collection were chart entries closest to LVAD implantation and cardiac transplantation. Post-LVAD data reflect device settings clinically indicated and represent the combined effects of native LV function as well as LVAD-related unloading.

### Quantitative Proton Nuclear Magnetic Resonance Spectroscopy

0.1 to 0.4 g of snap-frozen cardiac tissue was weighed out from each patient sample and homogenized in a mortar grinder in the presence of liquid nitrogen. Ice cold perchloric acid (PCA, 4 mL, 12%) extraction was carried out as described previously allowing for protein precipitation and separation of hydrophilic and lipophilic metabolites [16]–[18] The samples were centrifuged, and after the aqueous phase was neutralized with KOH, they were centrifuged again. The extract was lyophilized overnight. Next, the dry pellets were redissolved in 0.55 mL of deuterium oxide (D_2_O) for hydrophilic extracts and with 1.5 ml of deuterated methanol/chloroform for lipids.

H-NMR was carried out as published previously in order to evaluate cardiac metabolism [19]. In short, one-dimensional magnetic resonance spectra of hydrophilic and lipid extracts were recorded on a Bruker DRX500 MHz NMR spectrometer and processed with TopSpin software (Bruker, Billerica, MA). A 5-mm TXI inverse probe was used for all experiments. The operating frequency for proton NMR was 500 MHz, and a standard presaturation pulse program was used for water suppression. Other parameters included: 40 accumulations, 90-degree pulse angle, 0-dB power level, 7.35-µs pulse width, 10-ppm spectral width, and 12.85-s repetition time. Absolute quantification of metabolites (µmol/g tissue) was performed using trimethylsilyl propionic-2,2,3,3,-d_4_ acid (0.6 mM/L)as an external standard. ^1^H chemical shifts of spectra were referenced to trimethylsilyl propionic-2,2,3,3,-d_4_ acid at 0 ppm.

Metabolites in cardiac tissue were compared via t-test (for comparisons of non-failing and failing hearts) and paired t-test (for comparisons of pre-LVAD vs. post-LVAD samples) using SAS/STAT® software version 9.2, The SAS Institute, USA.

## Results

Patient characteristics are found in Table 1. There were no statistically significant demographic differences between the failing/post-LVAD group and the non-failing group. Patients in the failing and post-LVAD group had a mean age of 40, and those in the non-failing group were on average 48 years old.

**Table 1:** Demographic Characteristics of Patients.

	Failing and Post-VAD (n = 8)	Non-Failing (n = 8)
Patient Characteristics		
Mean age (years)	40.5±11.5	48±8.2
Male gender	7 (88%)	3 (38%)
Caucasian	4 (50%)	7 (88%)
African American	4 (50%)	0 (0%)
Hispanic	0 (0%)	1 (13%)
Mean duration of HF prior to LVAD (months)	56±22	
Days on LVAD	143±41	
Baseline Cardiac Index (l/min/m^2^)	1.8±.4	
Baseline Pulmonary Capillary Wedge Pressure	27±7	
HeartMate XVE LVAD*	5 (62%)	
HeartMate II LVAD*	3 (39%)	
Medical Therapy/Interventions Pre-LVAD		
Intravenous inotropic agent	8 (100%)	
Intravenous vasodilator	5 (62%)	
β-blocker	0 (0%)	
ACE inhibitor/ARB	2 (25%)	
Aldosterone antagonist	7 (88%)	
Diuretic	8 (100%)	
Medical Therapy During LVAD Support		
Intravenous inotropic agent	0 (0%)	
β-blocker	6 (75%)	
ACE inhibitor/ARB	7 (88%)	
Aldosterone antagonist	7 (88%)	
Diuretic	6 (75%)	

HF = Heart Failure, LVAD = Left Ventricular Assist Device, NYHA = New York Heart Association, ACE = Angiotensin-Converting Enzyme, ARB = Angiotensin II Receptor Blocker.

* Thoratec, Pleasanton, CA.

Pre-LVAD, all patients in the failing/post-LVAD group were on intravenous inotropic agents and diuretics, 88% were on an aldosterone antagonist, 62% and intravenous vasodilator, and 25% an ACE inhibitor. During LVAD support 88% of patients in this group were on an aldosterone antagonist and/or an ACE inhibitor. 75% took a β-blocker and/or a diuretic prior to LVAD implantation (Table 1). Seven of the eight donor patients received IV vasopressors (unknown doses) after brain death, but none of these donor patients received inotropic agents. Table 1 lists clinical and hemodynamic information for hearts in the failing/LVAD group only. All parameters improved significantly after LVAD implantation.

Table 2 describes the baseline characteristics of the failing heart group. 5 (62.5%) had the HeartMate XVE® device implanted, while 3 (37.5%) of the patients had the HeartMate II® implanted. Patients in this study were on LVAD support an average of 143±41 days. Ejection fraction improved significantly after LVAD implantation from 10.0 percent (sd 1.07) to 25.6 percent (sd 11.4), (p = 0.003). Cardiac index also improved significantly after LVAD implantation from 1.5 l/min/m^2^ (sd 0.4) to 2.47 l/min/m^2^ (sd 0.7), (p = 0.016).

**Table 2:** Clinical and Hemodynamic Variables for Failing Hearts.

	Failing	Post-VAD (matched n = 8)	p-value
Ejection Fraction (%)	10 (1.0)	26 (11.0)	0.0065
LVEDs (cm)	6.8 (1.2)	4.1 (1.3)	<0.0001
LVEDd (cm)	7.6 (1.2)	4.9 (1.4)	<0.0001
Mean PAP (mmHg)	40.1 (8.4)	19.0 (6.2)	0.0062
MAP (mmHg)	72.4 (6.0)	90.1 (11.7)	0.0155
NYHA Heart Class	4 (0)	2.1 (1)	<0.0001

LVEDs: Left Ventricular End Systolic Diameter, LVEDd: Left Ventricular End Diastolic Diameter, PAP: Pulmonary Artery Pressure, MAP: Mean Arterial Pressure, NYHA: New York Heart Association.

Key differences in myocardial metabolism are described in Table 3. In general, amino acids were significantly lower in failing than in non-failing hearts and did not significantly increase post-LVAD (Table 3). Alanine levels were higher in non-failing hearts (4.88±1.44 µmol/g of tissue vs. 2.13±1.57; p = 0.0004) than in failing hearts; and did not significantly increase post-LVAD support (2.83±1.6; p = 0.116). Levels of aromatic amino acids were higher in non-failing hearts (10.8 ±1.64 µmol/g of tissue) vs. failing hearts (8.29±2.8; p = 0.018); and did not increase post-LVAD (7.19±3.23; p = 0.330). Glutamine was higher in non-failing hearts (7.46±1.64 µmol/g of tissue) than in failing hearts (4.74±1.99; p = 0.0048) and did not increase post-LVAD (5.22±0.53; p = 0.442).

**Table 3:** Metabolite Differences Between Patient Groups (µmol/g of tissue).

Metabolite Category	Non-Failing	Failing	Post-VAD
Glucose/Sugars			
Glucose	1.49±0.54	0.72±0.61*	1.46±0.61**
Lactate	11.2±3.69	5.72±5.76*	15.6±5.54**
Amino Acids			
Glutamine	7.46±1.64	4.74±1.99*	5.22±0.53
Alanine	4.88±1.44	2.13±1.57*	2.83±1.6
Aromatic Amino Acids	10.8±1.64	8.29±2.80*	7.19±3.23
Other			
Total Creatine	10.4±1.97	4.70±3.32*	5.20±3.4
Succinate	3.55±0.91	2.19±1.23*	3.48±1.25

Metabolite differences between non-failing and failing hearts (* = p<0.05) and failing and post-VAD hearts (** = p<0.05).

Glucose was higher in non-failing hearts (1.49±0.54 µmol/g of tissue) than in failing hearts (0.72±0.61 µmol/g of tissue; p = 0.0037), and the level increased again significantly after LVAD support (1.46±0.61 µmol/g of tissue; p = 0.0302). Lactate also was higher in non-failing hearts (11.2±3.69 µmol/g of tissue) than in failing hearts (5.72±5.76 µmol/g of tissue; p = 0.0045); and also increased again significantly after LVAD support (15.6±5.54 µmol/g of tissue; p = 0.0082).

Total creatine was higher in non-failing (10.4±1.97 µmol/g of tissue) than failing hearts (4.70±3.32 µmol/g of tissue; p = 0.00009); and creatine did not increase significantly after LVAD support (5.20±3.4 µmol/g of tissue; p = 0.3554). Succinate was significantly higher in non-failing (3.55±0.91 µmol/g of tissue) than failing hearts (2.19±1.23 µmol/g of tissue; p = 0.0113), and did not change significantly post-LVAD (3.48±1.25 µmol/g of tissue; p = 0.0917).

## Discussion

Our data indicates that significant changes in myocardial metabolism occur in the failing heart and some, but not all of these changes, are improved by LVAD placement. The amino acid levels in this study indicate that these substrates decrease significantly in cardiac tissue from non-failing to failing hearts and they do not recover during LVAD support. These data seem to support the hypothesis that the heart is depleting its amino acid stores and that unloading the heart through LVAD support does not allow replenishment of these losses.

**Figure 1:**
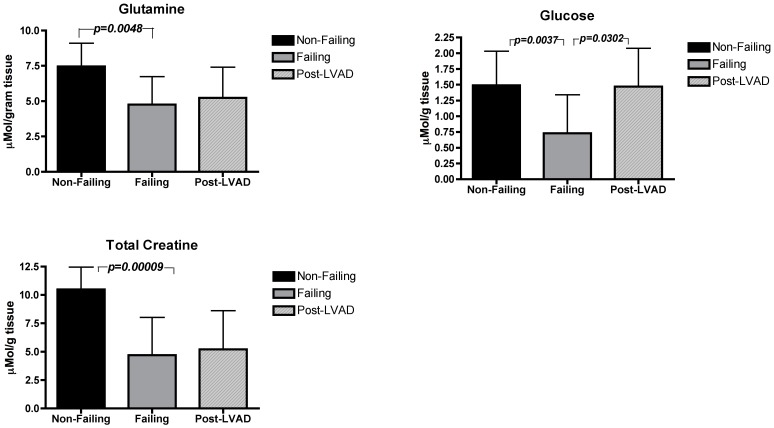
Differences in select metabolites between non-failing, failing, and post-LVAD hearts. P-values indicated only where differences are statistically significant (p<0.05).

Select metabolites from each category are represented graphically in Figure 1. We chose to measure glutamine levels as a result of previous data showing the cardioprotective effects of this amino acid. Glutamine has been shown to return contractile function *in vitro* in chick cardiomyocytes post ischemia/reperfusion (I/R) injury [20]. Glutamine has also been shown to enhance myocardial tissue metabolism, glutathione content, and improve myocardial function (cardiac output) after I/R injury in a working heart model [21]. This particular amino acid has also been shown in double-blind, placebo-controlled, randomized trials carried out by our group and others to be protective of the myocardium in human clinical trials when administered during cardiopulmonary bypass (CPB) [22], [23]. In these studies, glutamine treatment prior to CPB decreased troponin I [22], creatine kinase-MB [23], and clinical complications [23]. It has been shown that the most dominant pathway through which glutamine exerts its effects on the cardiomyocyte and other cells in the body is through the induction of heat shock proteins [21]. Additional important pathways exist including glutamine conversion to alpha ketoglutarate to be utilized in the TCA cycle.

Substrates related to glucose metabolism showed a different pattern pre-/post-LVAD placement than the measured amino acid levels. Both glucose and lactate decreased in heart failure but increased again after LVAD support. It is known that during heart failure there is a switch in substrate preference from fatty acids (β-oxidation) to carbohydrates (glycolysis) as the primary fuel preference. This is part of the reversion to the fetal energy substrate pattern known to occur during heart failure [24]. Unlike amino acid metabolism, levels of substrates related to carbohydrate metabolism seem to revert back to normal after LVAD support. It is plausible that the glucogenic amino acids are being utilized to synthesize glucose and to maintain glycolysis thus causing amino acid stores to “burn out” while carbohydrate levels are replenished in the post-LVAD samples. However, more focused studies need to be carried out to prove this hypothesis.

Total creatine levels (creatine plus phosphocreatine) were measured in an attempt to determine relative energy availability in the cardiac tissues. The pattern of total creatine data looked much like the amino acid data as it decreased significantly from non-failing to failing hearts and remained low post-LVAD. This was interesting in light of our overall hypothesis, as there is a link in the literature between glutamine and creatine. In rat models of ischemia/reperfusion, glutamine supplementation leads to an increase in the creatine and phosphocreatine content of the myocardium [21], [25]. While patients in this study were not supplemented with glutamine, it is not surprising that creatine followed a similar overall pattern to glutamine levels as these previous data showed.

Succinate levels were also measured in order to determine the function of the citric acid cycle (CAC) under the disease states studied. Levels of CAC intermediates are crucial for the oxidation of acetyl-CoA to CO_2_ and the generation of NADH and FADH_2_ for the electron transport chain. Increasingly cardiovascular researchers are studying the role of ‘anapleurosis’ in the heart, meaning the process of replenishing depleted metabolic cycle intermediates (in this case CAC intermediates) [26]. The fact that succinate levels were significantly decreased in heart failure and did not improve significantly post-LVAD indicates that CAC intermediates are affected in the failing heart, and that they follow the same pattern as the amino acids, and creatine.

Increasing anapleurotic flux in the setting of cardiac disease is considered beneficial [26]. One challenge in accomplishing this increase is that activity of the enzymes aconitase and citrate synthase are decreased in heart failure [27]. For this reason, carbohydrates and fatty acids are utilized less efficiently than substrates that bypass these enzymes and enter further along the TCA cycle. Thirteen of the 22 amino acids enter the CAC past this point, one is glutamine.

In conclusion, these data show that cardiac tissue amino acid stores are depleted in heart failure and they do not recover after LVAD support. This is of particular interest for the amino acid glutamine since it is known to have cardioprotective effects. Total creatine and succinate (a CAC intermediate) follow the same pattern as amino acids, while metabolites related to glycolytic metabolism recover their levels after LVAD implantation. It is known that individuals who have lower BMI's (and thus less lean body mass) have higher mortality from heart failure. In light of the obesity paradox and the cardiac data presented here, a clinical trial of targeted amino acid supplementation in patients with heart failure should be considered.

There are several limitations to this study. A disadvantage of measuring metabolites only in cardiac tissue is the inability to obtain samples from the vast majority of patients with heart failure. Our study is confined to patients with advanced disease who received mechanical cardiac support. We did not prospectively collect peripheral blood on these patients so it is unknown if these changes in tissue metabolic substrates correlate with peripheral abnormalities. It is also unknown if these changes effect patients with less severe disease. It is interesting to note that data from the Framingham study has recently shown that higher peripheral glutamine is strongly associated with reduced cardiovascular risk. [28] Our results suggest only that these abnormalities involve the myocardium in end-stage heart failure. Further prospective studies are necessary to evaluate the correlation of peripheral and tissue metabolites as well as their prognostic significance. These experiments are also limited in that they only measure cardiac metabolites and as such provide limited insight into the molecular mechanisms of these processes. Future combined studies in metabolomics, transcriptomics and proteomics will be necessary to elucidate the full mechanisms of these abnormalities as well as overall system biology of heart failure. Finally, there is lack of data from the donor patients and donor hearts including BMI, diabetes status, and catecholamine levels. Procurement of donor heart tissue is the only manner in which to reasonably obtain non-failing control tissue. These limitations are universal when utilizing human myocardial samples. End-stage heart failure is a complex phenotype. Given the small sample size these findings should be considered hypothesis generating until the underlying mechanisms are elucidated and the findings confirmed in larger cohorts.

SAS and all other SAS Institute Inc. product or service names are registered trademarks or trademarks of SAS Institute Inc. in the USA and other countries.
